# The Order of the Police in Reference to the Privileges of Physicians in Crossing Chicago Bridges

**Published:** 1866-10

**Authors:** 


### THE ORDER OF THE POLICE IN REFERENCE TO THE PRIVILEGES OF PHYSICIANS IN CROSSING CHICAGO BRIDGES.

We are certain the public as well as the medical practitioners of Chicago will be grateful to the Captain of our police force for his very humane and reasonable order by which physicians, to a certain extent, have precedence in crossing the bridges over Chicago river.

Those of our readers, who have visited Chicago, must have observed how often and how long carriages are obliged to wait at the dozen or more swing bridges for the numerous vessels which pass on our river.

It was no uncommon occurrence for physicians to find themselves detained for half an hour at the end of a line of carriages extending an eighth of a mile, thus being obliged to wait not only for the vessels to pass through, but for the vehicles of every description, before them to cross, however pressing their calls might be.

At present, a physician has the privilege of going immediately "to the front" and having precedence in crossing.

Physicians will thus be saved much anxiety and loss of time; the people generally, who have sick friends, will be spared many an hour of anxious waiting for their physicians, detained by the ever-swinging bridges.

We repeat, the order of the police is a humane one, of which the public as well as physicians will recognize the propriety.

